# Effects of internal iliac artery embolization on systemic inflammatory response syndrome in dogs with simulated-pelvic-fracture combined with massive bleeding

**DOI:** 10.1186/s40779-016-0085-2

**Published:** 2016-04-27

**Authors:** Bing Xie, Ming Liang, Da-Peng Zhou, Wen Zhao, Jing-Yang Sun, Jing-Jing Rong, Jing Tian

**Affiliations:** Department of Orthopedics, General Hospital of Shenyang Military Region, Shenyang, 110016 China; Department of Cardiology, General Hospital of Shenyang Military Region, Shenyang, 110016 China

**Keywords:** Internal iliac artery embolization, Systemic inflammatory response syndrome, Interventional treatment cabin

## Abstract

**Background:**

Pelvic fracture combined with massive bleeding (PFCMB) is a complex issue in clinical practice. Currently, the use of angiography and embolization for the treatment of PFCMB obtains good results. The aim of this study is to observe the effects of early internal iliac artery embolization on the SIRS in dogs with simulated-pelvic-fracture combined with massive bleeding.

**Methods:**

Twenty adult dogs were randomly divided into an embolization group (EG) and a control group (CG). For the two groups, heart rate, respiratory rate and body temperature and other physiological variables were measured, and IL-6, TNF-α and arterial blood gas levels were monitored. These variables were assayed every 30 min until death in the CG, while dogs in the EG underwent arterial angiography after 60 min of modeling. The internal iliac artery was embolized on the injured side.

**Results:**

The average time to SIRS in the CG was 3.56 h, occurring at a rate of 90 % (9/10) within 24 h, with a mortality rate of 50 % (5/10); the average time to SIRS for the EG was 5.33 h, occurring at a rate of 30 % (3/10) within 24 h, with a mortality rate of 10 % (1/10). When SIRS occurred in the EG, the mean plasma IL-6 level was 52.66 ± 7.38 pg/ml and the TNF-α level was 11.45 ± 2.72 ng/ml, showing a significant difference with those of the CG (*P* < 0.05). In the two groups, the respiratory rate and leukocyte levels were higher at each monitored time after modeling than those before modeling; the mean arterial pressure, levels of hemoglobin and oxygen partial pressure were significantly lower at each time point after modeling than those before modeling except for the mean arterial pressure at 0 h in EG; the platelet levels at 4 and 8 h were higher than those before modeling; and the differences were statistically significant (*P* < 0.05). In the EG, the mean arterial pressure, heart rate, respiratory rate and hemoglobin levels at 2 , 4 and 8 h were lower than those at 0 h; the levels of leukocytes, platelets and carbon dioxide partial pressure at 4 and 8 h after modeling were higher than those at 0 h, and the differences were statistically significant (*P* < 0.05, *P* < 0.01); in the CG after modeling, the mean arterial pressure, levels of hemoglobin and carbon dioxide partial pressure at 2, 4 and 8 h were lower than those at 0 h; the levels of heart rate and leukocytes were higher than those before modeling; the respiratory rate and platelet levels at 4 and 8 h were higher than those at 0 h; and the differences were statistically significant (*P* < 0.05). The levels of the mean arterial pressure and hemoglobin at 4 and 8 h and the pH values at 8 h after modeling in the EG were significantly higher than those in the CG, while the heart rate and respiratory rate at 4 and 8 h were significantly lower than those in the CG. The pH values at 8 h after modeling were significantly lower than those of the other monitored times in the CG (*P* < 0.05, *P* < 0.01). The two groups had elevated levels of alkaline phosphatase after injury induction.

**Conclusion:**

Through the use of an on-spot interventional treatment cabin, early internal iliac artery embolization can control bleeding associated with pelvic fractures, delay the occurrence of SIRS, and improve the success rate of the treatment of pelvic fracture combined with bleeding.

## Background

Pelvic fractures combined with massive bleeding (PFCMB) are most often caused by high-impact injuries. In such cases, wounded individuals are severely injured, with high mortality, due to death from hemorrhagic shock and multiple organ dysfunction syndromes (MODS). Systemic inflammatory response syndrome (SIRS) is a type of stress response of the immune system after the body is exposed to severe trauma, blood loss and other factors. SIRS is most common following the early stages of injury, and once the balance between SIRS-driven inflammatory responses and anti-inflammatory responses is broken, MODS occurs. Therefore, the earlier that surgeries are implemented to delay or control SIRS, the higher the success rates of the treatment of PFCMB.

Currently, the use of angiography and embolization for the treatment of PFCMB has good results. However, because of the limitations of associated with some medical conditions, most hospitals do not implement such strategies in emergency situations. Following large scale catastrophes such as earthquakes, wars and other situations, pelvic fractures occur in large quantities; however, early intervention or damage control surgery are rarely carried out due to difficulties in casualty evacuations and on-site medical conditions.

This study investigates how the early implementation of the internal iliac artery embolization, through on-spot interventional treatment to control pelvic-fracture bleeding, delays SIRS and improves success rates in the treatment of PFCMB.

## Methods

### Experimental animal grouping

This study was approved by the General Hospital of the Shenyang Military Region Council on Animal Care in accordance with the guidelines of the Ministry of Science and Technology of the People’s Republic of China (The Guidance of Experimental Animal Welfare, 2006).

Twenty healthy adult dogs, with body weights 15 ± 2 kg, provided by the Experimental Animal Division, Shenyang Military General Hospital, were randomly divided into an embolization group (EG, *n* = 10) and a control group (CG, *n* = 10). Tracheal intubation of the unilateral carotid artery was carried out under general anesthesia, and the blood pressure, heart rate, respiration and ECG were monitored using a multi-channel polygraph.

### Experimental animal procedures

A customized mold was used to fix each dog’s pelvis at a side laying position. Next, a 20 kg hammer fell from a height of 1 m to produce a moderate pelvic fracture. Puncture angiography was performed in the right femoral artery of the model animals, with a bifurcation of 5 cm between the iliac and the external iliac arteries, and a 21st surgical dagger was used to mutilate the internal iliac artery per cutem at the flat area of the femoral head.

### Monitoring indicators & SIRS standards

The heart rates (HR), respiratory rates (RR) and body temperatures were measured, and venous blood and arterial blood were collected after anesthesia from the 2 groups. Blood analyses and L-6 and TNF-α tests of the venous line, arterial blood gases, routine blood tests, liver and renal functions, and blood gas analysis tests were also carried out. The CG was monitored every 30 min after induction of the model until death, while arterial angiography and embolization of the injured side of the internal iliac artery of the EG was implemented after 60 min of the onset of the model injury. SIRS was determined to occur after any 2 of the following 5 indicators occurred: 1) the heart rate increased by 50 % more than average, 2) the respiratory rate increased by 100 % more than average, 3) the WBC count increased by 100 % or decreased by 50 % compared to the average baseline, 4) the PaCO_2_ decreased by 25 % more than the average, and/or the 5) temperature increased or decreased by 1 °C from the average.

### Detection methods for IL-6 and TNF-α

Venous blood samples (>4 ml) were collected and centrifuged to separate the plasma, which was then analyzed by enzyme-linked immunosorbent assay (ELISA). ELISA kits (DiroCHEK®, Synbiotics Corporation, San Diego, CA) were used following the manufacturer’s instructions. The wavelength of the microplate reader was 450 nm and the reference wavelength was 620 nm for the determination of optical density.

### Statistical analyses

SPSS 17.0 Statistical Software was used to analyze the data, with the results expressed as the mean ± SD. Each set of data measurements first underwent tests for homogeneity of variance. The samples with homogeneity of variance next underwent single factor analysis of variance, and the others underwent Tamhane's test. The different time points within the same group were analyzed using repeated measures of comparison and were considered to have a significant difference with *P* < 0.05.

## Results

**Fig. 1:**
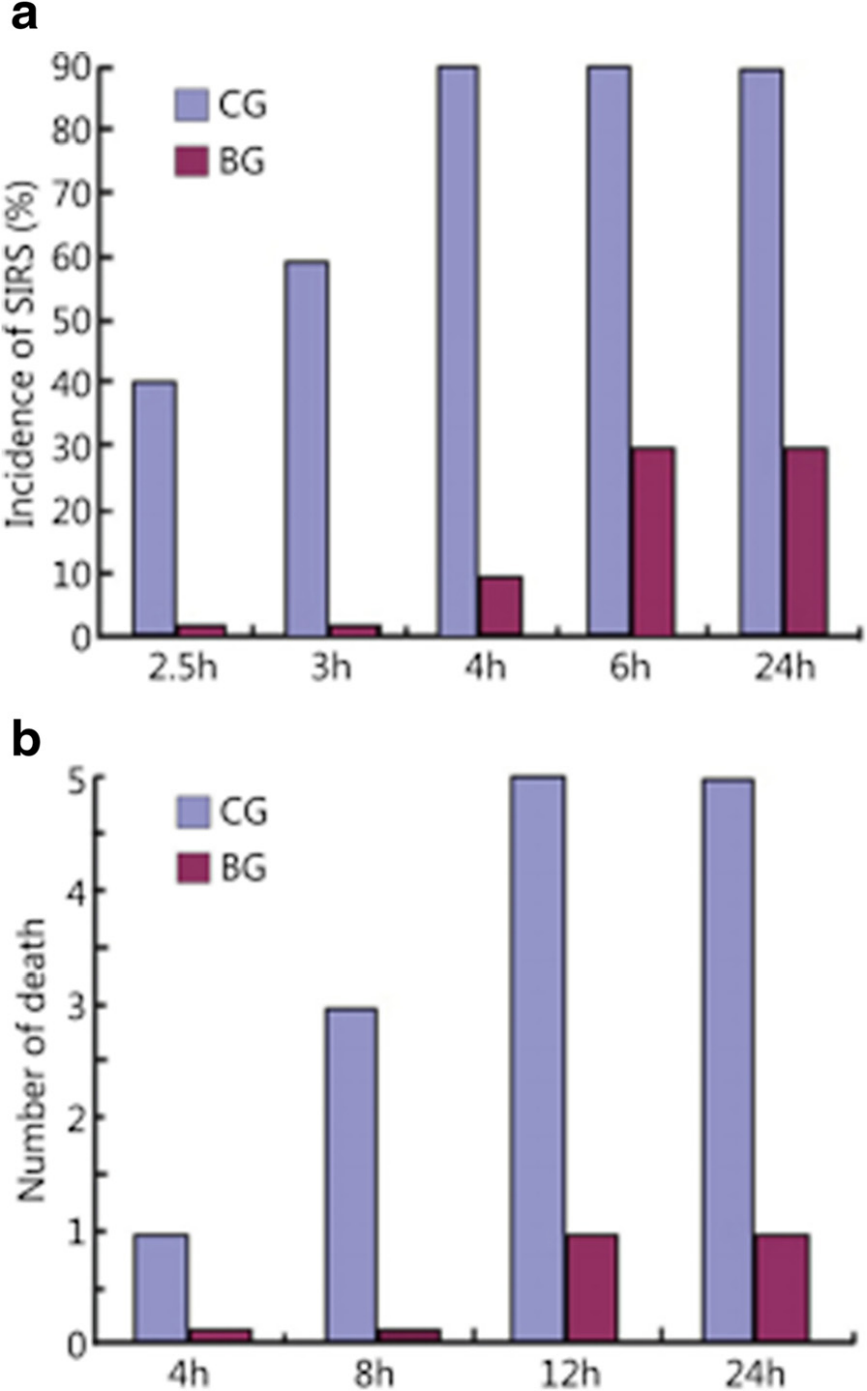
Incidence of SIRS and mortality within 24 h for the two groups. **a** Incidence of SIRS within 24 h between the 2 groups. **b** Mortality of SIRS animals within 24 h between the 2 groups

SIRS occurred in 4, 2 and 3 of the dogs in the CG after 2.5, 3 and 4 h of injury, respectively, with an average time of 3.56 h, and the incidence rate was 90 % (9/10) within 24 h; 1, 2 and 2 of the CG animals died 4, 8 and 8–24 h after injury, respectively, with a mortality rate of 50 % (5/10). On the contrary, SIRS occurred in 1 and 2 of the EG dogs 4 and 6 h after modeling, at an average time of 5.33 h at a rate of 30 % (3/10) within 24 h; 1 died 12 h death after onset of the model injury, with the mortality rate of 10 % (1/10). SIRS and death did not occur in the other dogs within 24 h (Fig. 1).

When SIRS occurred, the mean plasma IL-6 levels and TNF-α levels of the EG were 52.66 ± 7.38 pg/ml and 11.45 ± 2.72 ng/ml, respectively, showing a significant difference with 23.03 ± 6.11 pg/ml and 6.79 ± 1.17 ng/ml of the CG.

In the two groups, the respiratory rates and the leukocyte levels were higher at each time point after injury than those before injury. The mean arterial pressures, levels of hemoglobin and oxygen partial pressure were significantly lower at each time point after injury than those before injury except for mean arterial pressure at 0 h in the EG. The platelet levels at 4 and 8 h were higher than those before injury, and the differences were statistically significant (*P* < 0.05). In the EG after injury, the mean arterial pressures, HRs, respiratory rates and hemoglobin levels at 2, 4 and 8 h were lower than those at 0 h. The levels of leukocytes and platelets and carbon dioxide partial pressure at 4 and 8 h after injury were higher than those at 0 h, and the differences were statistically significant (*P* < 0.05, *P* < 0.01). In the CG, after injury, the mean arterial pressures, levels of hemoglobin and carbon dioxide partial pressures at 2, 4 and 8 h were lower than those at 0 h, and the heart rate and leukocyte levels were higher than those before injury. The respiratory rates and platelet levels at 4 and 8 h were higher than those at 0 h, and the differences were statistically significant (*P* < 0.05). The levels of mean arterial pressures and hemoglobin at 4 and 8 h and the pH values at 8 h after injury in the EG were significantly higher than those in CG, while the heart rates and respiratory rates at 4 and 8 h were significantly lower than those in the CG. The pH values at 8 h after injury were significantly lower than those at other time points in the CG (*P* < 0.05, *P* < 0.01). The two groups had elevated levels of alkaline phosphatase after injury (Tables 1, 2, 3 and 4).

**Table 1:** Comparisons of vital signs between the two groups

Group		MAP (mmHg)	HR (t/min)	RR (t/min)
CG	Pre-modeling	145.2 ± 11.2	190.5 ± 9.7	16.7 ± 3.5
	0 h post-modeling	139.5 ± 9.6	210.9 ± 11.4	35.5 ± 5.4
	2 h post-modeling	110.4 ± 8.5	229.7 ± 10.5	38.5 ± 3.6
	4 h post-modeling	97.2 ± 7.5	230.2 ± 9.7	39.2 ± 2.8
	8 h post-modeling	85.2 ± 10.3	260.5 ± 11.5	42.6 ± 7.3
EG	Pre-modeling	139.6 ± 16.1	201.1 ± 10.2	17.9 ± 2.9
	0 h post-modeling	142.7 ± 8.5	217.8 ± 11.2	38.2 ± 9.4
	2 h post-modeling	117.2 ± 9.6	210.5 ± 7.9	27.5 ± 5.5
	4 h post-modeling	115.6 ± 5.3^*^	195.8 ± 5.8^*^	29.3 ± 9.6^*^
	8 h post-modeling	102.8 ± 5.8^*^	200.6 ± 11.3^*^	25.5 ± 6.9^*^

The data are expressed as the mean ± SD

*CG* Control group, *EG* Embolization group, *MAP* Mean arterial pressure, *HR* Heart rate, *RR* Respiratory rate

^*^ *P* < 0.05 compared with CG group at the same time point; *P* < 0.05 results of repeated measures data of ANOVA are *F*_MAP_ (embolization) =12.61 and *F*_RR_ (embolization × time) =20.52

**Table 2:** Blood indices comparisons between the two groups

Index	Group	Pre-modeling	Post-modeling			
			0 h	2 h	4 h	8 h
WBC	CG	4.2 ± 2.1	12.7 ± 3.4	17.8 ± 5.1	18.6 ± 9.3	19.1 ± 9.1
	EG	4.4 ± 1.7	11.8 ± 5.7	13.6 ± 7.2	14.7 ± 8.2	14.9 ± 6.3
	*F*	1.52	2.81	1.99	1.28	2.08
	*P*	0.43	0.17	0.22	0.65	0.21
HGB	CG	152.2 ± 12.6	129.1 ± 6.2	102.3 ± 1.2	92.1 ± 4.5	84.7 ± 9.2
	EG	148.1 ± 9.7	131.3 ± 4.5	121.4 ± 6.3	112.6 ± 7.1	101.2 ± 8.1
	*F*	1.69	1.89	5.56	17.22	8.29
	*P*	0.29	0.26	0.06	0.0003	0.008
PLT	CG	188.4 ± 11.7	185.5 ± 7.1	192.4 ± 9.2	290.5 ± 6.5	257.1 ± 4.5
	EG	189.9 ± 16.3	189.5 ± 9.3	190.4 ± 11.3	291.4 ± 8.7	272.3 ± 11.7
	*F*	1.94	1.71	1.51	1.79	5.76
	*P*	0.24	0.28	0.38	0.25	0.06

The data are expressed as the mean ± SD. *P* < 0.05 results of repeated measures data of ANOVA are *F*_HGB_ (embolization × time) = 164.77, *F*_HGB_ (time) = 12.61, and *F*_PLT_ (embolization) = 7.33

*CG* Control group, *EG* Embolization group, *WBC* White blood cell, *HGB* Hemoglobin, *PLT* Blood platelet

**Table 3:** Blood gas analysis comparison between the two groups

Index	Group	Pre-modeling	Post-modeling			
			0 h	2 h	4 h	8 h
pH value	CG	7.34 ± 0.04	7.29 ± 0.06	7.27 ± 0.03	7.22 ± 0.04	7.16 ± 0.03
	EG	7.32 ± 0.03	7.33 ± 0.05	7.27 ± 0.04	7.25 ± 0.04	7.22 ± 0.03
	*F*	1.78	1.44	1.78	1.11	8.58
	*P*	0.25	0.41	0.25	0.79	0.0067
PaO_2_	CG	120.4 ± 5.7	112.4 ± 6.9	100.5 ± 5.4	108.5 ± 6.1	111.3 ± 7.1
	EG	121.6 ± 6.1	114.6 ± 7.2	105.2 ± 7.7	111.5 ± 4.7	109.3 ± 6.4
	*F*	1.15	1.09	2.03	1.68	1.23
	*P*	0.78	0.82	0.22	0.29	0.62
PaCO_2_	CG	40.5 ± 3.8	47.6 ± 5.6	35.2 ± 6.1	38.6 ± 5.9	43.3 ± 5.7
	EG	41.2 ± 5.7	42.8 ± 3.7	40.7 ± 5.4	45.6 ± 6.1	45.8 ± 6.3
	*F*	2.25	2.29	1.28	1.07	1.22
	*P*	0.19	0.19	0.65	0.82	0.66

The data are expressed as the mean ± SD. *P* < 0.05 results of repeated measures data of ANOVA are *F*_PH-value_(embolization) = 54.18, *F*_PH-value_(time) = 6.71, *F*_PH-value_(embolization × time) = 95.24, and *F*_PaCO2_ (time) = 32.45

*CG* Control group, *EG* Embolization group

**Table 4:** Comparison of levels of alkaline phosphatase between the two groups

Group	0 h	2 h	4 h	8 h	24 h
CG	57.1 ± 3.1	128.5 ± 6.2	169.3 ± 5.7	238.5 ± 7.1	264.2 ± 6.7
EG	56.9 ± 4.3	77.6 ± 3.4	82.3 ± 2.9	149.6 ± 3.3	168.7 ± 2.8
*F*	3.12	6.29	6.97	7.57	8.22
*P*	0.15	0.04	0.02	0.01	0.005

*CG* Control group, *EG* Embolization group

The data are expressed as the mean ± SD. *P* < 0.05 results of repeated measures data of ANOVA, *F*(embolization) = 14.37, *F*(time) = 81.14, and *F*(embolization × time) = 232.45

## Discussion

### Reasons for PFCMB

Pelvic fracture combined with massive bleeding is a common primary cause of patient death. In high-impact injuries, nearly 15–30 % of patients show hemodynamic instability [1], and approximately 67.9 % of such patients do not stabilize their blood pressures, even with pelvic straps, anti-shock trousers, external fixation, sufficient blood transfusion and other measures [2]. The bleeding sources for pelvic fractures include arterial bleeding, venous plexus bleeding and fracture hemorrhage. The two latter types of bleeding can reach a relatively stable status at the point of fracture, and the retroperitoneal pressure of the fractures can increase to a certain degree to form a physiological filling to stop bleeding, or the situation can improve after the arterial bleeding has been effectively controlled. On the other hand, arterial bleeding is often difficult to stop on its own, especially in “open-book” fractures, as the pelvic volume is further expanded to generate the “vacuum aspiration” phenomenon, leading to continuous bleeding, which can eventually cause hypovolemic shock and even death. Slater and Barron [3] summarized the relationship between the locations of pelvic fractures and damaged blood vessels and revealed that the piriformis can cut sharply into the fascia of the greater sciatic foramen, leading to a tearing of the superior gluteal artery; fractures in the inferior pubic bone or in the sciatic hole can lead to injuries of the pudendal artery, while in the upper part of obturator, fractures in the superior pubic ramus and acetabulum can easily lead to injuries of the obturator artery.

### Bleeding is one of the major drivers of SIRS in the early stages of trauma

In 1991, the American College of Chest Physicians & Society of Critical Care Medicine (ACCP/SCCM) proposed the concept of SIRS and explained it to be the result of the imbalance of bodily inflammatory reactions and anti-inflammatory responses, hypothesizing that this imbalance would inevitably lead to MODS [4]. Bleeding is one of the hallmarks that change cytokine activities after the trauma, resulting in excessive inflammation and a “second hit” to the body. Acute blood loss or shock would then activate the monocyte-macrophage system and cause injuries of vascular endothelial cells, which could both lead to a massive release and activation of proinflammatory cytokines. Therefore, early increases in inflammatory cytokines are mainly due to organ ischemia and hypoxia caused by blood loss, while the middle and late stages are mainly caused by interactions of cytokines. Once the balance of proinflammatory and antiinflammatory responses of these cytokines is tipped, SIRS occurs and eventually leads to MODS.

### The role of IL-6 and TNF-α levels in SIRS diagnoses

SIRS is a condition of neuro-immune and metabolic systems and is co-mediated by a variety of endogenous cytokines, and cytokine blood concentrations are often used to indicate the degree of SIRS. After trauma, macrophage phagocytosis and the antigen-presenting capacity of the body decreases, while the secretion of IL-6, TNF-α and other inflammatory cytokines increases [5]. IL-6 and TNF-α are important cytokines in the body's own immune restoration network and can limit the expansion of injuries and inflammation and provide a suitable micro-environment for tissue healing and repair *in vivo* as well as drive an excessive inflammatory response, thereby increasing damage to the body.

IL-6 is an important marker after severe trauma, reflecting the severity of injury. High concentrations of IL-6 can promote endothelial cells to express intercellular adhesion molecule (ICAM), increase the adhesion between leukocytes and endothelial cells, and lead to the microvascular obstructions. Therefore, some researchers have speculated that IL-6 may be used in the diagnosis of traumatic shock [6, 7]. IL-6 has a strong proinflammatory activity and can act directly on vascular endothelial cells, increasing their permeability and promoting differentiation and the production of antibodies by β-cells, and by inducing and regulating the synthesis of acute phase proteins which further modulate the immune response, hematopoiesis and bodily defense.

TNF-α is a proinflammatory cytokine released early after trauma that can activate a cascade of cytokine reactions and induce the synthesis of interleukins and secondary inflammatory mediators such as arachidonic acid metabolites, oxygen free radicals and lipid peroxidation materials, among others [8]. After trauma, the moderate secretion of TNF-α supports anti-infection processes and the repair of damaged tissue, while too much TNF-α or an improper balance of cytokines may lead to secondary inflammatory damage such as fever, shock or tissue organ hemorrhage and necrosis [9].

### The potential for early intervention of PFCMB by embolization

#### Early intervention by embolism clearly may improve treatment success rates

Heetveld *et al*. [10] proposed that, with the exclusion of abdominal organ injuries or already-stabilized patients with abdominal organ injuries, angiography should be carried out within 45 min of reaching the emergency room after the non-invasive fixation of a pelvic fracture, to determine whether large blood vessel injuries are present and whether embolization could be used to control pelvic bleeding. Agolini *et al*. [11] analyzed factors influencing treatment outcomes and concluded that embolization within 3 h after the injury could significantly improve the survival rate of patients with PFCMB. Currently, the clinical applications of angiography and embolization for the treatment of PFCMB also achieve good results, and the reported success rate is approximately 85–100 % [12]. Balogh *et al*. [13] carried out early interventions by embolization in PFCMB patients, and the mortality decreased from 35 to 7 %; accordingly, angiography should be the preferred diagnosis and treatment option if there are no other life-threatening injuries [14, 15].

#### The Rich vascular network in pelvis ensure the safety of embolization

Blood vessels in the pelvic area make a number of broad connections and often support bleeding after pelvic fracturing, but they also provide the anatomical basis for the safe treatment of blood vessels by embolization. Through the study of bodies after death, some researchers have confirmed that the branches of the internal iliac artery, the internal iliac artery itself and the external iliac artery have wide connections. There are also connections among the upper branch of the superior gluteal artery and the sacral lateral artery, the hip artery, the lateral sacral artery and the middle sacral artery, the inferior gluteal artery and the obturator artery pudendal artery branch and the ascending branch of the femoral external carotid artery. The two branch ligaments around the spine of the inferior gluteal artery also have connections. Therefore, it is thought that the selective and non-selective embolization of the internal iliac artery serves as a reliable, safe and effective means of promoting hemostasis [16, 17]. Some researchers have also proposed that embolization of the internal iliac artery might lead to the ischemic necrosis of the gluteal muscle [18]. In a more recent study, Andrew *et al*. [19] further confirmed that if the injuries permitted, selective iliac artery embolization would not cause the necrosis of the gluteal muscle. Velmahos *et al*. [20] researched 30 cases of temporary occlusion of the internal iliac artery, and there were no apparent complications. Therefore, the unilateral or bilateral internal iliac artery occlusion surgery in pelvic fractures may be considered to be a safe and effective means of treatment [21, 22].

Despite early embolization improving the treatment success rate of PFCMB [23, 24], Morozumi *et al*. [25] also obtained satisfactory results using a mobile imaging system for PFCMB in the emergency room. Although most clinicians have accepted this treatment concept, there are few hospitals that can carry out embolization within 3 h following injury. Pinto *et al*. [26] confirmed that multi-slice CT can also help to locate bleeding pelvic vessels, while in special cases such as earthquakes and military combat, distance would make it impossible to implement CT scanning as well as early intervention surgeries in the emergency room. This study revealed that early intervention could control bleeding, delay SIRS, and stabilize vital signs for a longer time. Therefore, we developed an on-spot interventional treatment cabin for such treatments of the wounded.

The on-spot interventional treatment cabin is a part of a “quick medical service support system for use in military battle”, with the characteristic advantages of high mobility and independence. We believe that it can provide the following advantages in the field for the interventional examination and embolization of pelvic fractures: 1) an earlier opportunity to carry out damage control operations, helping to improve the success rates; 2) sterile conditions for reducing the probability of infections after surgery; 3) the mobility of the cabin, which enables surgery to be carried out anytime and anywhere; 4) the ability of the cabin to maintain constant temperature and protect the supplies, water and electricity, enabling operations free from outside interference; 5) the comprehensive abilities of the cabin, which include complete on-spot vascular interventional imaging and treatment, one-stop diagnosis of multiple fractures by the C-arm X-ray machine and the first aid care of critical cases through the use of monitoring and emergency equipment.

## Conclusions

This study shows that early internal iliac artery embolization can control pelvic fracture bleeding, delay the occurrence of SIRS, and improve the success rate in the treatment of pelvic fracture combined with bleeding through the use of an on-spot intervention treatment cabin.

## Abbreviations

ACCP/SCCM: American College of Chest Physicians & Society of Critical Care Medicine
CG: control group
CT: computed tomography
ECG: electrocardiogram
EG: embolization group
ELISA: enzyme-linked immunosorbent assay
HGB: hemoglobin
HR: heart rate
ICAM: intercellular adhesion molecule
IL-6: Interleukin-6
MAP: mean arterial pressure
MODS: multiple organ dysfunction syndromes
PFCMB: pelvic fracture combined with massive bleeding
PLT: blood platelet
RR: respiratory rate
SIRS: systemic inflammatory response syndrome
TNF-α: tumor necrosis factor-alpha
WBC: white blood cell
